# Circuit change during extracorporeal membrane oxygenation: single-center retrospective study of 48 changes

**DOI:** 10.1186/s13054-023-04503-9

**Published:** 2023-06-02

**Authors:** Thibaut Genty, Stanislas Burguburu, Audrey Imbert, Calypso Roman, Wirth Camille, Jacques Thès, François Stéphan

**Affiliations:** 1 Cardiothoracic Intensive Care Unit, Hôpital Marie LannelongueGroupe Hospitalier Paris Saint Joseph, 133 Avenue de La Résistance, 92350 Le Plessis-Robinson, France; 2 Department of Anesthesiology, Extracorporeal Circulation Referral Center, Marie Lannelongue Hospital, Groupe Hospitalier Paris Saint Joseph, Le Plessis-Robinson, France; 3 School of Medicine, University Paris-Saclay, Le Kremlin-Bicêtre, France; 4 INSERM U999, Pulmonary Hypertension: Pathophysiology and Novel Therapies, Hôpital Marie Lannelongue, Groupe Hospitalier Paris Saint Joseph, Le Plessis-Robinson, France

**Keywords:** ECMO, Oxygenator change, Bleeding complication, Oxygenator thrombosis, Transfusion

## Abstract

**Background:**

Bleeding and thrombosis induce major morbidity and mortality in patients under extracorporeal membrane oxygenator (ECMO). Circuit changes can be performed for oxygenation membrane thrombosis but are not recommended for bleeding under ECMO. The objective of this study was to evaluate the course of clinical, laboratory, and transfusion parameters before and after ECMO circuit changes warranted by bleeding or thrombosis.

**Methods:**

In this single-center, retrospective, cohort study, clinical parameters (bleeding syndrome, hemostatic procedures, oxygenation parameters, transfusion) and laboratory parameters (platelet count, hemoglobin, fibrinogen, PaO_2_) were collected over the seven days surrounding the circuit change.

**Results:**

In the 274 patients on ECMO from January 2017 to August 2020, 48 circuit changes were performed in 44 patients, including 32 for bleeding and 16 for thrombosis. Mortality was similar in the patients with vs. without changes (21/44, 48% vs. 100/230, 43%) and in those with bleeding vs. thrombosis (12/28, 43% vs. 9/16, 56%, *P* = 0.39). In patients with bleeding, numbers of bleeding events, hemostatic procedures, and red blood cell transfusions were significantly higher before vs. after the change (*P* < 0.001); the platelet counts and fibrinogen levels decreased progressively before and increased significantly after the change. In patients with thrombosis, numbers of bleeding events and red blood cell transfusions did not change after membrane change. No significant differences were demonstrated between oxygenation parameters (ventilator FiO_2_, ECMO FiO_2_, and PaO_2_) and ECMO flow before vs. after the change.

**Conclusions:**

In patients with severe and persistent bleeding, changing the ECMO circuit decreased clinical bleeding and red blood cell transfusion needs and increased platelets and fibrinogen levels. Oxygenation parameters did not change significantly in the group with thrombosis.

## Background

Extracorporeal membrane oxygenation (ECMO) is a recognized treatment for refractory hypoxemia (veno-venous ECMO, VV-ECMO) and cardiogenic shock (veno-arterial ECMO, VA-ECMO) [1]. Bleeding is common during ECMO (34% of patients on VV-ECMO and 44% on VA-ECMO), whereas thrombosis is rarer (11% on VV-ECMO and 7% on VA-ECMO) [2]. Bleeding and thrombosis induce major morbidity and mortality [2], and their prevention is a major treatment goal. Recent guidelines by the Extracorporeal Life Support Organization (ELSO) state that anticoagulation should be used for most patients whose anti-factor Xa (antiXa) level is within the 0.3–0.7 IU/mL range [3]. Thrombosis can require replacement of the membrane oxygenator [3]. Although performed in clinical practice, changing the membrane is not mentioned in any guidelines as part of the management of bleeding [4].

The objective of this study was to evaluate the course of clinical, laboratory, and transfusion parameters after ECMO circuit changes warranted by bleeding or thrombosis.

## Methods

This retrospective study included patients managed in the 23-bed adult intensive care unit of the Marie Lannelongue Cardiothoracic Surgery Hospital (Groupe Hospitalier Paris Saint Joseph, Le Plessis-Robinson, France). A national review board CERAR approved the study (IRB0001025). All patients received an information and no-objection statements in accordance with the French law on retrospective studies.

Records of consecutive adults managed with ECMO between January 2017 and August 2020 were screened to identify patients who required circuit changes due to bleeding or thrombosis with oxygenation failure. Each circuit change was handled as a separate event.

### Definitions

Bleeding group was defined as either bleeding persistent for more than two days or bleeding uncontrolled despite hemostatic procedures (arterio-embolization, surgical revision, packing, local hemostatic procedure during endoscopy). Bleeding localization was epistaxis or oral bleeding, cannula- or catheter-related bleeding, hemoptysis, and deep bleeding.

Thrombosis group included sudden thrombosis of head pump or circuit, clots on the membrane visible to the naked eye combined with hypoxemia defined as desaturation < 90% or right radial PaO_2_ < 80 mmHg with ECMO FiO_2_ ≥ 80%. When oxygenator did not allow direct visualization of clots on the membrane, thrombosis was defined as membrane dysfunction with a post-oxygenator blood gas sample showing an ECMO PaO_2_/FiO_2_ < 200 mmHg [5].

### ECMO monitoring

For VV- and VA-ECMO, unfractionated heparin (UFH) was administered to maintain antiXa between 0.2 and 0.4 IU/mL, with dosage adjustments after each antiXa assay performed at least every 12 h. In compliance with ELSO recommendations, in patients with bleeding, UFH was interrupted for up to 12 h or as long as needed to control the bleeding [3]. Once the bleeding was controlled, effective anticoagulation was reintroduced in patients with formal indications (e.g., pulmonary embolism, loss of arterial pulsatility, mechanical valve).

Laboratory tests performed at least twice daily included hemoglobin, platelet count, antiXa, fibrinogen, pH, PaCO_2_, and PaO_2_.

Protocolized transfusions of red blood cells (RBC), platelets, fresh frozen plasma (FFP), and fibrinogen were given to maintain hemoglobin > 7 g/dL, platelets > 30 000/mm^3^, fibrinogen > 1.5 g/dL, and prothrombin time > 50% [6].

### Data collection

For this study, we collected data over the seven days before and after the circuit change (from D-7 to D + 7). D-1 and D + 1 corresponded to the 24 h before and after the change. The data included the blood tests listed above, transfusion data (administered amounts of RBC units, FFP units, platelets, and fibrinogen), and technical data (ECMO and ventilator FiO_2_, ECMO flow rate). Clinical bleeding and hemostatic procedures were collected over the five days before and after the change (from D-5 to D + 5); this shorter collection period limited the risk of missing data.

### Statistical analysis

Data were analyzed using StatView 5.0 software (SAS Institute, Cary, NC). Data distribution was assessed by the Kolmogorov–Smirnov test. Normal data were described as mean ± SD and non-normal data as median [interquartile range]. Continuous variables were compared using Student's *t* test; for repeated measures, we performed ANOVA followed by the Scheffe test. *P* values < 0.05 were considered significant.

## Results

### Patients

We identified 274 patients managed with VA-ECMO (n = 211, 77%) or VV-ECMO (n = 63, 23%) during the study period. The proportion of patients with circuit changes was 18% (49 patients, 53 changes) overall and 31% (46 patients) among the 147 patients on ECMO for longer than seven days. We excluded five patients who were transferred on ECMO from another hospital, which wants to get back the ECMO device. For these patients, a scheduled change was performed. The remaining 44 patients had 48 circuit changes, including 32 (67%) for bleeding and 16 (33%) for thrombosis; two patients with bleeding had three changes each. The table reports the main patient characteristics and outcomes. Mortality was similar in the patients with vs. without circuit changes (21/44 (48%) vs. 100/230 (43%) P = 0.60) and in the patients with bleeding vs. thrombosis (Table 1).

**Table 1:** Characteristics and outcomes of the 44 patients with extracorporeal membrane oxygenation circuit changes

Variables	All (n = 44)	Bleeding^a^ (n = 28)	Thrombosis^b^ (n = 16)	*P* value
Number of circuit changes	48	32	16	–
Females, n (%)	15 (34)	9 (32)	7 (44)	0.72
Age, years, mean ± SD	55.0 ± 17.1	57.6 ± 15.7	51.5 ± 19.0	0.20
BMI, kg/m^2^, mean ± SD	28.2 ± 6.7	27.1 ± 7.4	29.4 ± 5.9	0.28
SAPSII points, mean ± SD	40.4.6 ± 11.9	37.9 ± 10.7	44.0 ± 12.0	0.17
Reason for ICU admission, n (%)				
*Surgical conditions*	32 (73)	19 (68)	13 (81)	0.34
Heart or lung transplantation	11	5	6	–
Heart surgery	11	7	4	–
Pulmonary thromboendarterectomy	5	3	2	–
ARDS after lung resection	5	4	1	–
*Medical conditions*	12 (27)	9 (32)	3 (19)	0.34
Cardiogenic shock	7	7	0	–
ARDS	5	2	3	–
Type of ECMO				
VV-ECMO	16 (36)	8 (29)	8 (50)	0.15
VA-ECMO	28 (64)	20 (71)	8 (50)	0.15
Type of oxygenator^c^				
HILITE 7000	17/48	13/32	4/16	0.29
QUADROX-PLS-I	16/48	11/32	5/16	0.83
EOS	15/48	8/32	7/16	0.19
Outcomes				
ICU stay, days, median [IQR]	31 [21–47]	32 [21–42]	31 [22–47]	0.94
Mechanical ventilation duration, days, median [IQR]	25 [15–40]	27 [15–38]	22 [16–43]	0.57
ECMO duration, days, median [IQR]	18 [13–26]	20 [12–26]	19 [12–28]	0.73
Renal replacement therapy, n (%)	20 (45)	11 (39)	9 (56)	0.28
Death, n (%)	21 (48)	12 (43)	9 (56)	0.39
During ECMO	15 (34)	9 (32)	8 (50)	0.24
After weaning off ECMO	5 (11)	3 (11)	2 (12)	0.85

*ECMO* extracorporeal membrane oxygenation, *VV* veno-venous, *VA* veno-arterial, *BMI* body mass index; *SAPS II* Simplified Acute Physiology Score version II, *ICU* intensive care unit; *ARDS* acute respiratory distress syndrome, *IQR* interquartile range, *SD* standard deviation

^a^The group with bleeding had 23 patients with pulmonary bleeding, 12 with epistaxis, 11 with deep bleeding, and 11 with cannula- or catheter-related bleeding; 16 patients required 23 hemostatic procedures: surgical revision (n = 6), intervention at bedside (n = 7), bronchial fiberoptic endoscopy (n = 5), arterial embolization (n = 3), upper gastrointestinal tract fiberoptic endoscopy (n = 2)

^b^In five patients, the reason for the circuit change was visibility to the naked eye of clots on the arterial side of the QUADROX-PLS-I® membrane; the post-oxygenator arterial blood gases were not evaluated. In four patients, the circuit was changed for a sudden thrombosis. The seven other patients had EOS or HILITE membranes that did not allow visual assessment of clots; membrane oxygenation dysfunction was diagnosed based on a post-oxygenator PaO_2_/FiO_2_ < 200 mmHg. The mean post-membrane ECMO PaO_2_/FiO_2_ in these seven patients was 101 mmHg

^c^Three types of ECMO device and three types of oxygenator were used during the study period: XENIOS (ex-MEDOS) pump (Fresenius, Bad Homburg, Germany) with a HILITE 7000 oxygenator (rheoparin coating); ROTAFLOW pump (Maquet, Rastatt, Germany) with a QUADROX oxygenator (heparin coating); and STOCKERT SCP pump (Livanova/Sorin, Milan, Italy) with an EOS oxygenator (phosphorylcholine coating)

### Group with bleeding (n = 32)

**Fig. 1:**
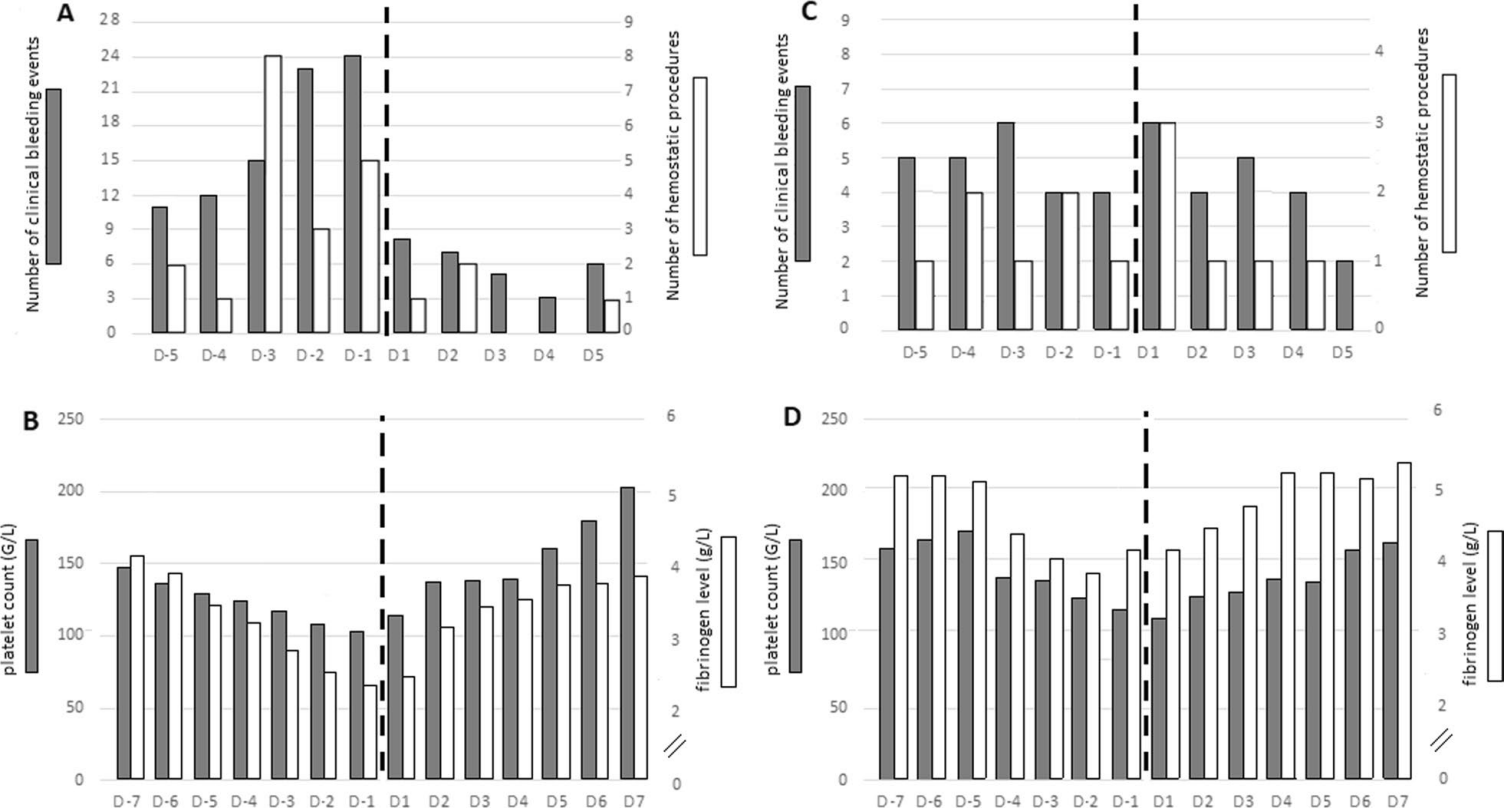
Main results in the group with bleeding (panels A and B) and in the group with thrombosis (panel C and D). **A** Group with bleeding: clinical bleeding events and hemostatic procedures during the five days before and the five days after the circuit change. The X-axis shows time in days; the black dotted line indicates the time of the circuit change. The Y-axis on the left shows the number of clinical bleeding events (gray bars) and the Y-axis on the right the number of hemostatic procedures (white bars). During the 5 days before the membrane change, 84 bleeding events occurred, 19 of which required a hemostatic procedure. In the 5 days following the membrane change, 29 bleeding events occurred, 4 of which required a hemostatic procedure. **B** Group with bleeding: changes in platelet counts and fibrinogen levels during the seven days before and the seven days after the circuit change. The X-axis shows time in days; the black dotted line indicates the time of the circuit change. The Y-axis on the left shows the platelet counts in G/L (gray bars) and the Y-axis on the right the fibrinogen levels in g/L (white bars). The ANOVA showed a significant difference in platelet (F = 5.7 P < 0.001) and fibrinogen values (F = 3.7 P < 0.001). In the period before the change (D-7 to D-1), the platelet values differed significantly (F = 10.2 P < 0.001). The decrease began at D-4 for a value of 34G/L P < 0.01. In the period after the membrane change (D + 1 to D + 7), the platelet values differed significantly (F = 7.2 P < 0.001). The increase started at D + 6 for a value of 73 G/L P = 0.01. In the period before the change (D-7 to D-1), the fibrinogen level differed significantly (F = 10.0 P < 0.001). The decrease began at D-3 for a value of 1.3 g/L P = 0.01. In the period after the membrane change (D + 1 to D + 7), the fibrinogen level differed significantly (F = 3.6 P = 0.002). The increase started at D + 5 for a value of 1.2 g/L P = 0.04. **C** Group with thrombosis: clinical bleeding events and hemostatic procedures during the five days before and the five days after the circuit change. The X-axis shows time in days; the black dotted line indicates the time of the circuit change. The Y-axis on the left shows the number of clinical bleeding events (gray bars) and the Y-axis on the right the number of hemostatic procedures (white bars). During the 5 days before the membrane change, 22 bleeding events occurred, 6 of which required a hemostatic procedure. In the 5 days following the membrane change, 24 bleeding events occurred, 7 of which required a hemostatic procedure. **D** Group with thrombosis: changes in platelet counts and fibrinogen levels during the seven days before and the seven days after the circuit change. The X-axis shows time in days; the black dotted line indicates the time of the circuit change. The Y-axis on the left shows the platelet counts in G/L (gray bars) and the Y-axis on the right the fibrinogen levels in g/L (white bars). The ANOVA showed a significant difference in platelet values (F = 2.0 P = 0.03). The ANOVA did not show a significant difference in fibrinogen level (F = 0.7 P = 0.71). In the period before the change (D-7 to D-1), the platelet values differed significantly (F = 2.9 P = 0.01). Individual comparison of platelet level showed no statistical difference. In the period after the membrane change (D + 1 to D + 7), the platelet values differed significantly (F = 2.8 P = 0.02). Individual comparison of platelet level showed no statistical difference. Before or after the membrane change, the fibrinogen values did not vary significantly (P = 0.12) Individual comparison of fibrinogen levels did not show any statistical difference

Compared to the five days before the circuit change, the five days after the change had significantly fewer clinical bleeding events (29 vs. 84, *P* < 0.001) and hemostatic procedures (4 vs. 19, *P* = 0.001) (Fig. 1A). Significant rises in fibrinogen (*P* < 0.001) and platelet (*P* < 0.001) levels occurred after the change (Fig. 1B), although the amounts of fibrinogen and platelets administered were not significantly different before and after the change (*P* = 0.31 and *P* = 0.11, respectively).

Compared to the pre-change period, the post-change period showed significant decreases in the numbers of administered RBC units (from 7.3 ± 6.4 to 3.7 ± 3.6, *P* = 0.01) and FFP units (from 3.0 ± 6.4 to 0.5 ± 1.4, *P* = 0.04). AntiXa level decreased after the change (from 0.20 ± 0.12 to 0.18 ± 0.10, *P* = 0.02).

Oxygenation did not improve after the change: ECMO FiO_2_, ventilator FiO_2_, PaO_2_, and ECMO flow and rotations per minute did not change significantly (data not shown).

### Group with thrombosis (n = 16)

Clinical bleeding events (22 vs. 24, *P* = 0.76) and hemostatic procedures (6 vs. 7, *P* = 0.78) did not differ before and after membrane change (Fig. 1C).

Comparing before and after the change, platelet level varied significantly (P = 0.03), while the fibrinogen level remained stable (P = 0.71) (Fig. 1D). Platelets and fibrinogen administered were not significantly different before and after the change (P = 0.65 and P = 0.70, respectively).

Compared with the pre-change period, the post-change period showed no significant decreases in the numbers of administered RBC units (from 3.2 ± 1.9 to 2.3 ± 1.7, P = 0.12) and FFP units (from 0.6 ± 1.4 to 0.2 ± 0.7, P = 0.37).

None of the oxygenation parameters improved significantly after the circuit change (ECMO FiO_2_, *P* = 0.82; ventilator FiO_2_, *P* = 0.34; and PaO_2_, *P* = 0.21).

## Discussion

The findings from this single-center retrospective study suggest that ECMO circuit changes in patients with persistent bleeding decrease the frequency of clinical bleeding events, the need for hemostatic procedures, and RBC and FFP transfusions. In contrast, in patients with thrombosis, such changes do not reduce the number of clinical bleeding events and hemostatic procedures or RBC and FFP transfusion. Oxygenation parameters did not improve in both groups after change. It is worth noting that our study was performed in a cardiothoracic surgery center and included over 70% of surgical patients mostly on VA-ECMO, whereas previous studies included only patients on VV-ECMO to treat acute respiratory distress syndrome.

Bleeding during ECMO is caused by several mechanisms that are dependent on ECMO duration. Over time, ECMO is associated with reductions in RBC deformability and with RBC clumping [7]. Blood in the circuit is subjected to high shear stress, which can cause hemolysis [8]. Finally, the increased consumption of fibrinogen and platelets also promotes bleeding [9]. The mechanisms of membrane oxygenator dysfunction may be interrelated, with thrombosis inducing hemolysis, which may in turn promote bleeding. The significant change in platelet levels in patients (thrombosis and bleeding) may be related to the physical characteristics of the circuit that play a role in platelet activation. Adherent platelets release their granules that contain proinflammatory cytokines and hemostatic factors [10]. Device-induced coagulation disorder is common, accounting for about 40% of circuit changes, but is accompanied with clinical bleeding in only half the cases [5].

The management of bleeding during ECMO is poorly standardized, with a consensus existing only for temporary withholding of anticoagulation [3]. The largest published cohort study (83 patients with at least one change) did not assess whether withholding anticoagulation decreased clinical bleeding [5]. Elevations in plasma-free hemoglobin (pfHb), serum lactate dehydrogenase (LDH), and D-dimer and declines in fibrinogen levels, platelet counts, and heparin doses may predict a need for a circuit change [5, 8]. However, D-dimer, pfHb, and LDH were not routinely measured in our center.

In the few patients with membrane thrombosis, hypoxemia persisted even after the membrane change. The lack of oxygenation improvement may have been related to a deterioration in patients lung function. Thus, the oxygenation might have worsened further, even if the circuits had not been change.

The main limitations of this study are the retrospective design and single-center recruitment of patients who underwent highly specific surgical procedures. Another limitation in the group with bleeding may be the exposure to heparin leached from the circuit over the weeks before the change but the day of the circuit change, the anticoagulation level remained low (median antiXa 0.08 IU/L [0–0.22]) despite some patients had formal indications for effective anticoagulation. The difference between pre- and post-membrane pressures was not collected, and we were therefore unable to accurately time the onset of membrane thrombosis.

## Conclusions

In patients with severe and persistent bleeding, changing the ECMO circuit decreased clinical bleeding and transfusion needs and increased platelet and fibrinogen levels. Oxygenation parameters were not significantly modified after the change in the group with thrombosis.

## Data Availability

All data can be accessed via the corresponding author.

## References

[1] Blum JM, Lynch WR, Coopersmith CM (2015). Clinical and billing review of extracorporeal membrane oxygenation. Chest.

[2] Thiagarajan RR, Barbaro RP, Rycus PT, Mcmullan DM, Conrad SA, Fortenberry JD (2017). Extracorporeal life support organization registry international report 2016. ASAIO J.

[3] McMichael ABV, Ryerson LM, Ratano D, Fan E, Faraoni D, Annich GM (2022). 2021 ELSO adult and pediatric anticoagulation guidelines. ASAIO J.

[4] Murphy DA, Hockings LE, Andrews RK, Aubron C, Gardiner EE, Pellegrino VA (2015). Extracorporeal membrane oxygenation-hemostatic complications. Transfus Med Rev.

[5] Lubnow M, Philipp A, Foltan M, Bull Enger T, Lunz D, Bein T (2014). Technical complications during veno-venous extracorporeal membrane oxygenation and their relevance predicting a system-exchange–retrospective analysis of 265 cases. PLoS ONE.

[6] Kortchinsky T, Mussot S, Rezaiguia S, Artiguenave M, Fadel E, Stephan F (2016). Extracorporeal life support in lung and heart-lung transplantation for pulmonary hypertension in adults. Clin Transplant.

[7] Lansink-Hartgring AO, Hoffmann R, van den Bergh W, de Vries A (2020). Changes in red blood cell properties and platelet function during extracorporeal membrane oxygenation. J Clin Med.

[8] Appelt H, Philipp A, Mueller T, Foltan M, Lubnow M, Lunz D (2020). Factors associated with hemolysis during extracorporeal membrane oxygenation (ECMO)-Comparison of VA- versus VV ECMO. PLoS ONE.

[9] Cartwright B, Bruce HM, Kershaw G, Cai N, Othman J, Gattas D (2021). Hemostasis, coagulation and thrombin in venoarterial and venovenous extracorporeal membrane oxygenation: the HECTIC study. Sci Rep.

[10] Millar JE, Fanning JP, McDonald CI, McAuley DF, Fraser JF (2016). The inflammatory response to extracorporeal membrane oxygenation (ECMO): a review of the pathophysiology. Crit Care.

